# An interesting finding of multiple calcified pulmonary nodules in a patient with rheumatoid arthritis

**DOI:** 10.1259/bjrcr.20150116

**Published:** 2015-09-16

**Authors:** Aws Alfahad, Paul Jennings, Simmon Smith, Nikta Niktash, John Curtin

**Affiliations:** ^1^ Radiology Department, Hull Royal Infirmary, Hull, UK; ^2^ Radiology Department, Ipswich General Hospital, Ipswich, UK; ^3^ Pathology Department, Ipswich General Hospital, Ipswich, UK; ^4^ Pathology Department, Norfolk and Norwich University Hospital, Norwich, UK

## Abstract

Calcified pulmonary (lung parenchymal) densities can occur in a number of conditions. A patient with rheumatoid arthritis presented with new right lung base nodules and left long base soft-tissue densities on his chest X-ray. These findings did not exist on his chest X-ray performed 2 years earlier. A subsequent thoracic CT scan showed multiple pleural-based irregular nodules of soft-tissue density that were partially calcified. There was also mediastinal and hilar lymphadenopathy. Following a discussion at the respiratory multidisciplinary team meeting, a CT-guided nodal biopsy was performed that showed necrotic lung tissue with palisaded histiocytes and fibrosis with chronic inflammation. No vasculitis or granulomata were seen and no there was evidence of malignancy. Appearances were consistent with a rheumatoid nodule. No mycobacteria or fungi were seen on Ziehl–Neelsen, Wade–Fite or periodic acid–Schiff stains. We concluded that this patient had unusual calcified rheumatoid lung nodules. Previously, calcified pulmonary nodules have been reported in the setting of Caplan’s syndrome in miners.

## Summary

Calcified pulmonary (lung parenchymal) densities can occur in a number of conditions. A patient with rheumatoid arthritis (RA) presented with new right lung base nodules and left lung base soft-tissue densities on his chest X-ray. These findings did not exist on his chest X-ray performed 2 years earlier. A subsequent thoracic CT scan showed multiple pleural-based irregular nodules of soft tissue density that were partially calcified. There was also mediastinal and hilar lymphadenopathy. Following a discussion at the respiratory multidisciplinary team meeting, a CT-guided nodal biopsy was performed that showed necrotic lung tissue with palisaded histiocytes and fibrosis with chronic inflammation. No vasculitis or granulomata were seen and no there was evidence of malignancy. Appearances were consistent with a rheumatoid nodule. No mycobacteria or fungi were seen on Ziehl–Neelsen, Wade–Fite or periodic acid–Schiff stains. We concluded that this patient had unusual calcified rheumatoid lung nodules. Previously, calcified pulmonary nodules have been reported in the setting of Caplan’s syndrome in miners.^1^

## Clinical presentation

A 63-year-old male, under the care of a rheumatologist for seropositive RA, had a chest X-ray requested by his general practitioner to investigate shoulder pain. This showed two nodules at the right lung base and a soft-tissue density at the left lung base. These findings were new compared with his prior chest X-ray performed 2 years ago.

## Differential diagnosis

The differential diagnosis for the partially calcified pulmonary nodules may include Wegener’s granulomatosis, metastatic pulmonary malignancy from osteosarcoma, chondrosarcoma, mucinous adenocarcinoma and thyroid cancer, amyloidosis, sarcoidosis, histoplasmosis, and tuberculosis.^2^ Confirmatory diagnosis thus may require a biopsy and pathological verification, as in this case.

## Medical history

At the time of presentation, the patient’s main complaint was pain and swelling in his left wrist with mild left shoulder pain. He had an occasional cough but no sputum production or haemoptysis. He was systemically well, with no fever or sweats. His weight was stable at 93 kg. His past medical history included mild asthma and a total hip replacement.

He was an ex-smoker with a 20 pack-year history. There was no known asbestos or silica exposure. He had Bacillus Calmette–Guerin vaccine (BCG) as a child. He had a family history of RA. He worked as a mechanic.

His medications included salbutamol, paracetamol and codeine phosphate. On examination, there were hand changes consistent with rheumatoid disease. His examination was otherwise normal.

## Laboratory findings

Rheumatoid factor (RF) was elevated (440), urate was normal, white cell count was elevated (15.9) and erythrocyte sedimentation rate (ESR) was elevated (46). His pulmonary function tests showed moderate airway obstruction with forced expiratory volume in the first second1 of 61%, forced vital capacity of 4.17 l and a transfer factor and coefficient 0.95 (70%). Multiple sputum cultures were negative for tuberculosis. He had a normal serum angiotensin converting enzyme (ACE) and a negative anti-nuclear cytoplasmic antibody (ANCA).

## Imaging findings

X-rays of both hands showed loss of radial crowding of the carpal bones, indicating a degree of subluxation typical of rheumatoid arthropathy. His chest X-ray showed right basal nodules and soft-tissue densities in the left lung base, which were new compared with the chest X-ray performed 2 years earlier when the patient was asymptomatic.

**Figure 1.**
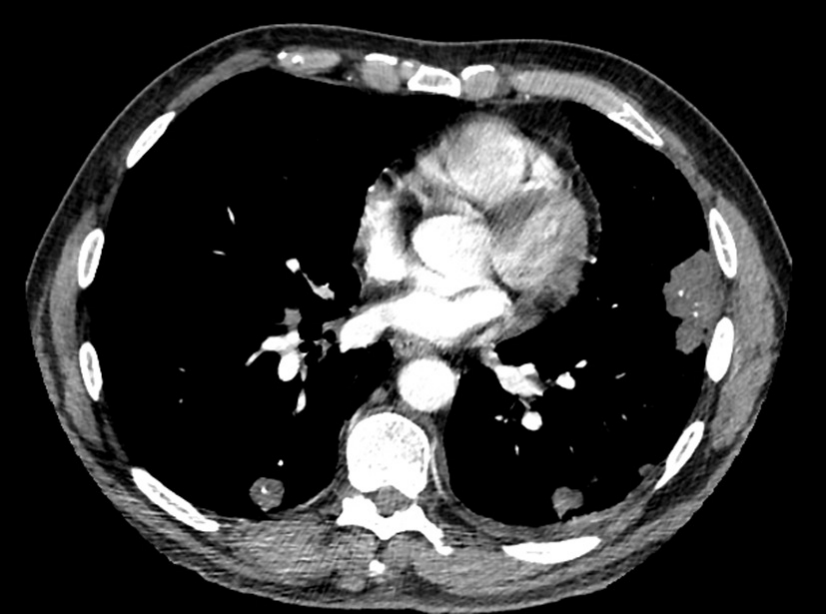
Axial CT image showing partially calcified pleural-based pulmonary nodules.

**Figure 2.**
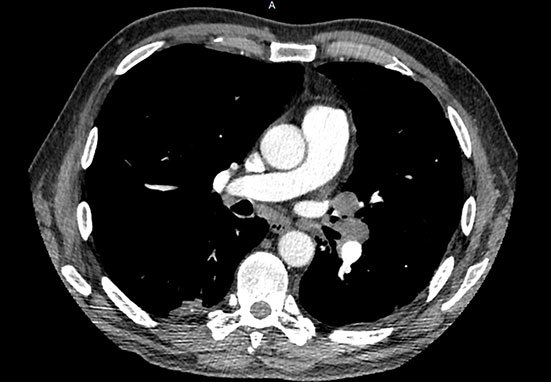
Axial CT image showing enlarged non-calcified hilar nodes and a partially calcified pleural-based pulmonary nodule.

A thoracic CT scan (Figures 1 and 2) showed multiple pleural-based irregular nodules ranging from 5 to 45 mm of mainly soft-tissue density that were partially calcified. There was an enlarged subcarinal node measuring 10 mm in minimum short-axis diameter and enlarged hilar nodes bilaterally measuring up to 17 mm. The abdominal CT scan was normal.

The MRI of the wrist revealed erosive changes, mainly at the proximal capitate with pronounced synovial proliferation as well as perilunate dislocation, which was attributed to his RA. After discussion at the lung multidisciplinary meeting, the patient proceeded to CT-guided lung biopsy.

## Histological findings

**Figure 3.**
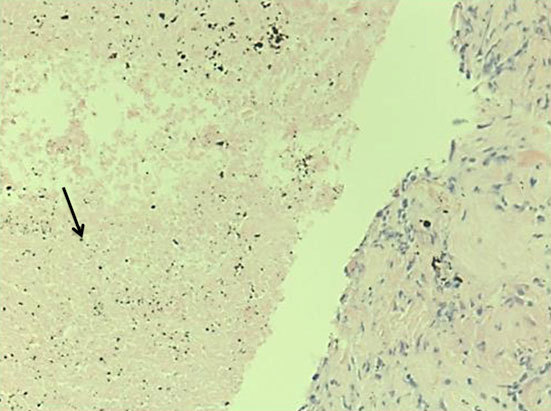
Histological core showing a necrobiotic centre (arrow) and containing anthracotic pigment.

**Figure 4.**
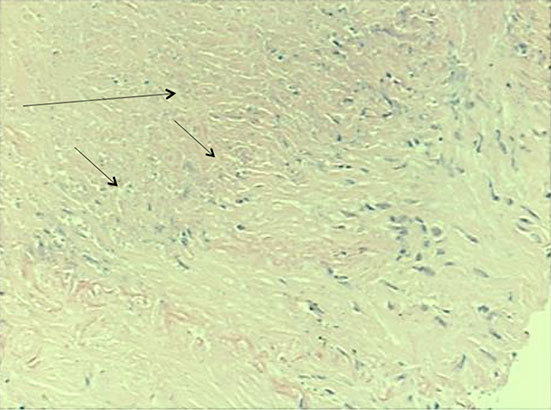
Histological core showing palisading histiocytes (short arrows) surrounding a zone of necrobiosis (long arrow).

Histological findings showed necrotic lung tissues with palisaded histiocytes at the edge, fibrosis and chronic inflammation (Figures 3 and 4). Neither vasculitis nor granulomata were seen and there was no evidence of malignancy. No mycobacteria or fungi were seen on Ziehl–Neelsen, Wade–Fite or periodic acid–Schiff stains. There was no silica or carbon in our specimen to suggest pneumoconiosis or Caplan’s syndrome. The appearances were suggestive of a rheumatoid nodule and pulmonary fibrosis.

## Management and follow-up

Our patient had a normal serum ACE with no skin or systemic manifestation, making a diagnosis of sarcoidosis unlikely. His inflammatory markers returned to normal and his ANCA was negative, making Wegner’s granulomatosis unlikely. The patient was systemically well with no fever, a negative tuberculosis sputum culture and biopsy results that excluded mycobacterial and fungal infection. There was no evidence of primary malignancy on the whole-body CT scan and the histological specimen showed no malignant cells.

The patient was treated as a case of seropositive RA. Sulfasalazine was chosen in view of his smoking history, lung function abnormalities and emphysema. There was remarkable improvement in his shoulder, wrist and hands joints pain after starting sulfasalazine. Clinically, he remained systemically stable with no respiratory symptoms. He was discharged from the respiratory clinic later in the year.

## Discussion

### Aetiology and demographics

RA is a chronic, immune-mediated illness in which polyarthritis is the most common presentation. It affects around 1% of the population.^3^ The peak age of onset is in the fifth decade of life. It affects females more commonly than males (2 : 1 to 3 : 1).^4^ The prevalence of pulmonary involvement ranges from less than 0.4% in radiological studies to 32%.^4^

Richman et al^5^ found the prevalence of extra-articular manifestation in Asian and Hispanic populations to be 21.5%; the most common manifestations were subcutaneous nodules (17.2%) and interstitial lung disease (3.6%).

Rheumatoid pulmonary nodules are more common in males, and usually occur in smokers with concomitant subcutaneous nodules and high titers of RF.^6^ Cavitating rheumatoid nodules without articular manifestations, though reported, are quite rare.^7^

Calcified pulmonary nodules have been reported previously in the setting of Caplan’s syndrome in miners.^1^ In 2007, Srinivas et al^8^ reported a case with multiple calcified pulmonary nodules in a female smoker with RA with no previous exposure to silica.

Pulmonary nodules in patients with RA were first described by Caplan in 1953, after his description of the characteristic pattern of multiple bilateral nodules on chest radiographs of coal miners with RA. On histological specimens, rheumatoid nodules appear round with a zone of palisading histiocytes perpendicular to the necrobiotic centre with adjacent vasculitis. These nodules have carbon and silica within the necrotic centre in Caplan’s syndrome.^9,10^

In our patient, there was no occupational exposure to silica or evidence of pneumoconiosis. Our histological specimen was typical of a rheumatoid nodule, with no evidence of silica or carbon particles.

The CT scan findings in our patient confirm the variable size and characteristic subpleural location of the rheumatoid nodules. The natural history of pulmonary nodules is variable and the nodules can remain static, increase in size and number and resolve or undergo malignant transformation.^11^ Some antirheumatic drugs such as methotrexate and leflunomide predispose to the development of pulmonary nodules.^12^ Lung nodules are usually asymptomatic and produce little pulmonary dysfunction but they can grow up to 7 cm in diameter and tend to rupture into the pleura, causing pneumothorax, hydropneumothorax or even pyopneumothorax.^13^ The unusual feature in our case was that many of the nodules had calcifications within them. Calcified rheumatoid nodules have usually been described in the setting of Caplan’s syndrome. To our knowledge, only one similar case has previously been reported.^8^ Our patient also had hilar and mediastinal lymphoadenopathy.^13^

Intrathoracic lymphadenopathy complicating rheumatoid lung is unusual. Khammassi et al^14^ report the case of a 51-year-old male with a history of RA who developed interstitial fibrosis with mediastinal and hilar adenopathy. Martinez et al^15^ claimed the first antemortem report of a patient with long-standing RA and interstitial lung disease who developed reactive mediastinal adenopathy coincident with increases in the activity of his interstitial process.^16^ Mediastinal adenopathy was discovered by means of a CT scan of the chest as part of an evaluation of interstitial lung disease. The use of better imaging techniques for this purpose will undoubtedly reveal more patients with this finding.

### Treatment and prognosis

Patients with RA live, on average, 3–12 years less than the general population.^16^ Current treatment approach to RA is to treat aggressively and as early as possible to prevent all the long-term morbidities and decrease the mortality associated with them.^3^ Early RA is defined anywhere from 3 months to 2–3 years of the disease.^17^ After a diagnosis of RA is made, all the patients should be started on a disease-modifying antirheumatic drug. Steroids also have some disease-modifying effect and hence contribute to overall disease control.^18^ Extra-articular manifestations are significant in prognostication, as they tend to correlate with mortality.^3^ Other poor prognostic factors include raised RF, serum anti-cyclic citrullinated peptide autoantibodies, ESR or C-reactive protein levels, and an increase in the number of joints involved and patient disability.^19^

## Learning points

Partially calcified pulmonary nodules can present in patients with RA who have not had occupational exposure to silica. Owing to the similarities between this condition and other immunological and pulmonary phenomena, clinicians must be vigilant to the large differential present when faced with a patient with similar presentation.
